# Association of cardiovascular health with the risk of dementia in older adults

**DOI:** 10.1038/s41598-022-20072-3

**Published:** 2022-09-19

**Authors:** Seunghoon Cho, Pil-Sung Yang, Daehoon Kim, Seng Chan You, Jung-Hoon Sung, Eunsun Jang, Hee Tae Yu, Tae-Hoon Kim, Hui-Nam Pak, Moon-Hyoung Lee, Boyoung Joung

**Affiliations:** 1 Division of Cardiology, Department of Internal Medicine, Severance Hospital, Yonsei University, 50-1 Yonsei-ro, Seodaemun-gu, Seoul, 03722 Republic of Korea; 2 Department of Cardiology, CHA Bundang Medical Center, CHA University, Seongnam, Republic of Korea; 3 Department of Biomedical Informatics, Yonsei University, Seoul, Republic of Korea

**Keywords:** Risk factors, Geriatrics, Disease prevention, Cardiology, Dementia, Alzheimer's disease

## Abstract

It has been becoming important to identify modifiable risk factors to prevent dementia. We investigated the association of individual and combined cardiovascular health (CVH) on dementia risk in older adults. From the National Health Insurance Service of Korea-Senior database, 191,013 participants aged ≥ 65 years without prior dementia or cerebrovascular diseases who had check-ups between 2004 and 2012 were assessed. Participants were stratified into three groups according to the number of optimal levels of CVH (low, 0–2; moderate, 3–4; and high CVH status, 5–6) and grouped by levels of individual CVH metrics, the number of optimal CVH metrics, and the CVH score. Over a median follow-up of 6.2 years, 34,872 participants were diagnosed with dementia. Compared with low CVH status, moderate and high CVH status were associated with a decreased risk of dementia (hazard ratio [95% confidence interval], 0.91 [0.89–0.92] for moderate; 0.78 [0.75–0.80] for high CVH status) including Alzheimer’s and vascular dementia. The risk of dementia decreased with an increase in the number of optimal CVH metrics (0.94 [0.93–0.94] per additional optimal metric) and with an increase in the CVH score (0.93 [0.93–0.94] per 1-point increase). After censoring for stroke, the association of CVH metrics with dementia risk was consistently observed. Among individual metrics, physical activity had the strongest association with the risk of dementia. In an older Asian population without prior dementia or cerebrovascular disease, a consistent relationship was observed between the improvement of a composite metric of CVH and the reduced risk of dementia.

## Introduction

Dementia is one of the greatest public health challenges in modern societies worldwide, including many developed countries^1,2^. Considering the absence of an effective treatment for dementia, identifying modifiable risk factors for dementia has become an important strategy to prevent dementia^3^.

The American Heart Association (AHA) has identified ideal cardiovascular health (CVH) metrics across seven established modifiable risk factors for cardiovascular disease (CVD) by defining optimal levels for each risk factor and emphasized the use of simple optimal CVH metrics for primordial prevention of CVD^4^. This simple tool consists of 4 behavioral metrics (smoking and ideal levels of body weight, physical activity, and diet) and 3 biological metrics (blood pressure [BP], fasting blood glucose, and total cholesterol) for the promotion of CVH^4^. Recent studies and meta-analyses have consistently revealed higher CVH (higher optimal CVH metrics) is associated with a substantially lower risk of death, coronary heart disease, and stroke^5–7^.

Given that some cardiovascular risk factors and mechanisms may have a common role in diseases affecting the heart and the brain, these CVH guidelines suggested by the AHA could have secondary benefits for the assessment of the risk and prevention of dementia^8–10^. However, the association of CVH metrics with the risk of dementia is still unclear due to the complexity. Moreover, how time-dependent CVH changes are related to the risk of dementia is also not well known, especially in an older population. The present study aimed to investigate the association of individual and combined CVH metrics, as cardiovascular risk factors, on the risk of dementia including Alzheimer’s disease (AD) and vascular dementia (VaD) in an older population.

## Methods

### Data collection and ethics declarations

Data were collected from the National Health Insurance Service of Korea (NHIS)-Senior database, which included data for 558,147 individuals recruited by a 10% simple random sampling method from a total of 5.5 million subjects aged ≥ 60 years in the National Health Information Database, which is an online-only database for qualified analysis centers, with formal payment and strict regulations on data release (https://nhiss.nhis.or.kr/)^11,12^. The NHIS incorporates all health-related data covering the entire population of the Republic of Korea. The NHIS-Senior database covered the following parameters: sociodemographic and socioeconomic information, insurance status, biennial health check-up examinations (including anthropometric measurements, laboratory investigations, and self-questionnaire surveys on health behaviors), and records of medical histories including prescription records^11,12^.

This study was approved by the Institutional Review Board of the Yonsei University Health System (4-2020-1387), and informed consent was waived. The study was conducted in compliance with the ethical guidelines of the Declaration of Helsinki. The NHIS-Senior database used in this study (NHIS-2016-2-171) was established by the NHIS in Korea.

### Study population and design

**Figure 1:**
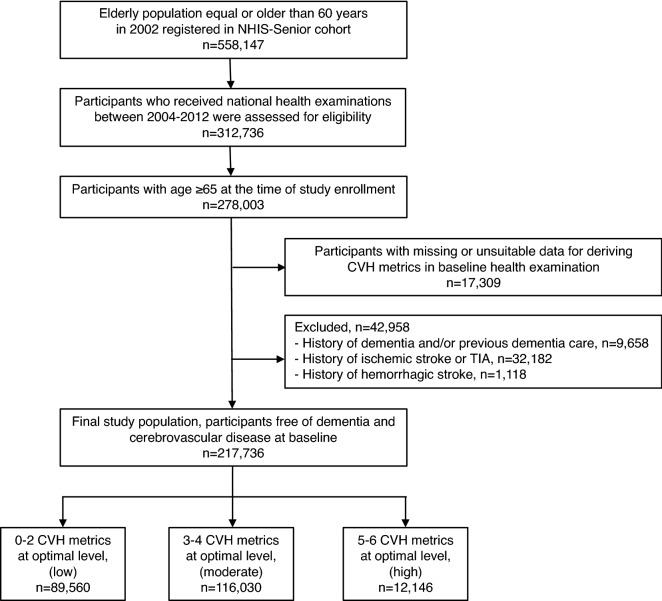
Flow-chart of study population selection. Abbreviations: *CVH* cardiovascular health, *NHIS* National Health Insurance Service of Korea, *TIA*, transient ischemic attack.

From the Korean NHIS-Senior database, 312,736 participants who underwent health examinations between 2004 and 2012 were selected, and medical records were reviewed until December 2014. Among them, a total of 278,003 participants aged 65 years or older were screened. The following participants were excluded: (i) with missing or unsuitable data for deriving CVH metrics in baseline health examination (n = 17,309); and (ii) with a prior history of any type of dementia (n = 9658), ischemic stroke or transient ischemic attack (n = 32,182), hemorrhagic stroke (n = 1118). Finally, we included 217,736 participants free of dementia or cerebrovascular diseases in the study analysis (Fig. 1). An outline of the study design is shown in Supplementary Fig. S1. There were a total of 5 study registrations with baseline health examinations at 2-year intervals for 8 years from 2004 to 2012.

### CVH metrics and CVH status

The CVH metrics employed in this study were based on the AHA cutoffs and criteria^4^. Dietary habits were not recorded during the data collection and therefore excluded from CVH metrics. Information on the other 6 metrics was obtained through routine health examinations and laboratory measurements. At each examination cycle, an interview and physical examination were conducted for each participant, and information on medical history was collected.

We categorized the 6 metrics (total cholesterol, fasting plasma glucose, BP, body mass index [BMI], smoking, and exercise) into 3 levels of poor, intermediate, and optimal, following the AHA’s recommendations (Supplementary Table S1). We used the sum of each optimal level of metric to calculate the number of optimal CVH metrics, ranging from 0 to 6. Then, we stratified the participants into three groups according to the calculated number of optimal metrics as follows: low (0–2 optimal metrics), moderate (3–4 optimal metrics), and high (5–6 optimal metrics) CVH status. We also derived the continuous 12-point CVH score by calculating the sum of scores assigned to each level of CVH metrics (poor = 0, intermediate = 1, and optimal = 2).

### Comorbidities and other covariates

To adjust for potential confounders except for diseases excluded at baseline according to exclusion criteria, we selected baseline sociodemographic factors, economic status based on health insurance premiums proportional to income, comorbidities, and medications that could be determinants of dementia incidence within the available database as covariates for the main analysis. We obtained information on selected comorbidities from inpatient and outpatient diagnoses in the NHIS-Senior database using the International Classification of Diseases-10th Revision (ICD-10) codes. Details of all the definitions and ICD-10 codes used for clinical outcomes, covariates, and comorbidities including diseases in the exclusion criteria are presented in Supplementary Table S2.

Some of the study participants (n = 11,273) were examed for depressive mood, lower extremity function, and cognitive function at baseline. The examination of depressed mood was screening participants who needed an accurate diagnosis and counseling for depression through questionnaires about their mood state. Cognitive function was examined using the activities of daily living scale and the Korean Dementia Screening Questionnaire (KDSQ) which consists of questions for global memory function and instrumental activities of daily living, including 5 items that can detect early changes in cognitive decline to diagnose dementia^13^. The KDSQ is not influenced by age or educational level and has shown a sensitivity of 0.79 and specificity of 0.80 for predicting dementia^13^.

### Assessment of dementia

We defined a diagnosis of dementia based on the relevant ICD-10 codes (F00 or G30 for AD, F01 for VaD, F02 for dementia with other diseases classified elsewhere, and F03 or G31 for unspecified dementia) and prescription of medication for dementia (rivastigmine, galantamine, memantine, or donepezil) in the records retrieved from NHIS claims data (Supplementary Table S2). When codes for both AD and VaD were recorded, we followed the principal diagnosis. The same participant could have one or more clinical events during the study duration, but only the first event of each outcome was considered. To evaluate the accuracy of our definition of dementia, a validation study was conducted at 2 hospitals with 972 patients, using the medical records of the patients and the results of cognitive function tests, and the positive predictive value was 94.7%^14–16^.

The time scale for the study outcomes was defined as the time in months between the time of study enrollment and the date of the first diagnosis with each type of dementia according to NHIS claims data. Participants without dementia were censored at the end of the observation period (end of the study period or death).

### Statistical analyses

Continuous variables are presented as mean ± standard deviation (SD) for normally distributed data or median (interquartile range [IQR]) for non-normally distributed data, and categorical variables are presented as number (percentage). Incidence rates and absolute rate differences of dementia according to CVH status are presented as events per 100 person-years (PY) at risk. Differences in the cumulative incidences between the three groups were evaluated using the log-rank test. We estimated the hazard ratios (HRs) of each type of dementia by CVH status, per 1 additional CVH metric at the optimal level, per 1-point increase in the continuous CVH score, and levels of individual components of CVH using time-varying Cox proportional hazard models. The levels of individual CVH metrics, CVH status, the number of optimal CVH metrics, and the CVH score, which changes at each examination conducted every 2 years during the study period, were used as time-varying variables for the analysis of dementia risk. In the sensitivity analyses, participants with interim stroke events were additionally censored at the date of stroke if they occurred before the end of the follow-up period. Unless otherwise noted, stroke censoring was performed in all results of the present study. We additionally assessed the risk of each type of dementia according to the number of optimal CVH metrics and the 12-point CVH score using the groups with the median of the number of optimal CVH metrics and the median of the CVH score as the reference groups.

CVH metrics already contain multiple anthropometric (BMI, BP) and laboratory (fasting blood glucose, total cholesterol) factors that are affected by underlying comorbidities such as hypertension, diabetes, obesity, and hyperlipidemia. Therefore, we excluded the presence or absence of these underlying comorbidities from the adjusted variables to avoid multi-collinearity or over-adjustment. All multivariable regression models were adjusted for age, sex, economic status, living area, comorbidities (atrial fibrillation, heart failure, myocardial infarction, coronary heart disease, peripheral artery disease, anemia, chronic kidney disease, hyperthyroidism, hypothyroidism, osteoporosis, sleep apnea, chronic obstructive pulmonary disease, chronic liver disease, and cancer), medications (oral anticoagulants, antiplatelet agents), depressive mood, lower extremity function, and cognitive function at the time of baseline examination. In the time-varying analysis of the association of dementia risk by the levels of individual CVH metrics, CVH components were mutually adjusted for each other in addition to all other covariates. All analyses were two-tailed, and *P* < 0.05 was considered statistically significant. All statistical analyses were performed using R (version 4.0.2; The R Foundation, www.R-project.org).

## Results

### Baseline characteristics

The comparison of baseline characteristics between the study groups according to baseline CVH status is summarized in Table 1. A total of 217,736 participants without a history of dementia or cerebrovascular diseases had data on all 6 CVH metrics at baseline. The mean ± SD age of participants was 71.7 ± 5.1 years, and 42.9% were male. Participants with lower baseline CVH status, denoting poor CVH, were more likely to be male, have low economic status, have more comorbidities including most cardiovascular diseases likely affecting dementia incidence except for some diseases, and take anticoagulant or antiplatelet agents than those with higher CVH status. The median (IQR) follow-up duration of overall dementia in the low, moderate, and high CVH status was 6.3 (4.0–7.7), 6.2 (3.8–7.7), and 5.4 (3.6–7.6) years, respectively.

**Table 1:** Baseline characteristics according to baseline CVH Status.

Characteristics	Low (0–2 optimal metrics), n = 89,560	Moderate (3–4 optimal metrics), n = 116,030	High (5–6 optimal metrics), n = 12,146	*P*-value^a^
Age, years	71.6 ± 5.0	71.9 ± 5.2	71.5 ± 4.9	< 0.001
**Sex**				< 0.001
Male	39,091 (43.6%)	49,209 (42.4%)	5,207 (42.9%)	
Female	50,469 (56.4%)	66,821 (57.6%)	6939 (57.1%)	
**Economic status**				< 0.001
Low	27,771 (31.0%)	35,558 (30.6%)	3521 (29.0%)	
Middle	20,050 (22.4%)	25,805 (22.2%)	2594 (21.4%)	
High	41,739 (46.6%)	54,667 (47.2%)	6031 (49.6%)	
**Living area**				< 0.001
Small city or rural area	54,112 (60.4%)	71,621 (61.7%)	7276 (59.9%)	
Metropolitan city	35,448 (39.6%)	44,409 (38.3%)	4870 (40.1%)	
**Comorbidities**				
Atrial fibrillation	2329 (2.6%)	2634 (2.3%)	205 (1.7%)	< 0.001
Heart failure	9784 (10.9%)	8550 (7.4%)	458 (3.8%)	< 0.001
Previous MI	2931 (3.3%)	2229 (1.9%)	112 (0.9%)	< 0.001
Coronary heart disease	3177 (3.5%)	2233 (1.9%)	88 (0.7%)	< 0.001
PAD	5038 (5.6%)	4374 (3.8%)	263 (2.2%)	< 0.001
Anemia	14,092 (15.7%)	23,605 (20.3%)	2982 (24.6%)	< 0.001
CKD	1558 (1.7%)	1384 (1.2%)	77 (0.6%)	< 0.001
Hyperthyroidism	2902 (3.2%)	3275 (2.8%)	323 (2.7%)	< 0.001
Hypothyroidism	2936 (3.3%)	3476 (3.0%)	360 (3.0%)	0.001
Osteoporosis	27,563 (30.8%)	36,479 (31.4%)	3769 (31.0%)	0.005
Sleep apnea	58 (0.1%)	52 (0.1%)	7 (0.1%)	0.151
COPD	8353 (9.3%)	9347 (8.1%)	882 (7.3%)	< 0.001
Chronic liver disease	22,475 (25.1%)	25,301 (21.8%)	2549 (21.0%)	< 0.001
Cancer	9993 (11.2%)	13,491 (11.6%)	1648 (13.6%)	< 0.001
**Medications**				
Warfarin	707 (0.8%)	745 (0.6%)	61 (0.5%)	< .001
DOAC	0 (0.0%)	0 (0.0%)	0 (0.0%)	1.000
Aspirin	25,090 (28.0%)	20,686 (17.8%)	921 (7.6%)	< 0.001
P2Y_12_ inhibitor	2247 (2.5%)	1476 (1.3%)	47 (0.4%)	< 0.001
Depressive mood^b^	2194 (52.9%)	3239 (53.3%)	550 (52.7%)	0.897
**Lower extremity function**^**b**^				
Gait disturbance during TUG^c^	108 (2.6%)	122 (2.0%)	11 (1.1%)	0.005
Time taken in TUG^c^ (s)	9.0 [7.0–10.0]	9.0 [7.0–10.0]	9.0 [7.0–10.0]	0.011
**Cognitive function**^**b**^				
Positive result in KDSQ^d^	823 (19.8%)	1290 (21.2%)	239 (22.9%)	0.055
KDSQ^d^ score	1.0 [0.0–3.0]	1.0 [0.0–3.0]	1.0 [0.0–3.0]	0.061
ADL scale^e^	6.0 [5.0–6.0]	6.0 [5.0–6.0]	6.0 [5.0–6.0]	0.097

Continuous variables are presented as mean ± standard deviation for normally distributed data or median [interquartile range] for non-normally distributed data, and categorical variables are presented as number (percentage).

^a^P-values for the contrast between three groups were derived from Pearson’s Chi-square for categorical variables and one-way analysis of variance or Kruskal–Wallis test for continuous variables where appropriate.

^b^Three additional tests, conducted on some of the study participants (n = 11,273) who underwent additional cognitive screening during the life transition period.

^c^TUG test measures the time that a person takes to rise from a chair, walk 3 m, turn around, walk back to the chair, and sit down. The more time taken indicates poorer physical function and balance.

^d^The KDSQ includes five items. Each item on the KDSQ is scored from 0 to 2, with a higher score indicating poorer function and a greater frequency. A KDSQ score ≥ 4 indicates a positive result.

^e^The ADL scale is a measurement of routine activities people do every day without assistance, which includes eating, bathing, getting dressed, and toileting, as well as mobility and continence. Higher scores indicate better and independent physical performance.

*ADL* activities of daily living, *CKD* chronic kidney disease, *COPD* chronic obstructive pulmonary disease, *CVH* cardiovascular health, *DOAC* direct oral anticoagulant, *KDSQ* Korean Dementia Screening Questionnaire, *MI* myocardial infarction, *PAD* peripheral artery disease, *TUG* timed up and go test.

In three additional screening tests, overall, there were no significant differences in the frequencies of depressive mood and in cognitive function between the three groups except for a few differences in lower extremity function (Table 1).

### Changes in the distribution of levels of individual CVH metrics

The changes in levels of individual CVH components according to CVH status at each health examination is shown in Supplementary Fig. S2. Higher CVH status represented a higher percentage of optimal levels in each component of CVH metrics. In the CVH metric of smoking, optimal levels accounted for a relatively high proportion of all three CVH status at each health examination when compared to other components of CVH metrics. In the CVH metric of physical activity, poor levels accounted for a relatively high proportion in the baseline health examination but with a particularly noticeable improvement throughout serial health examinations.

### Risk of dementia by CVH status

Over a median follow-up of 6.2 (IQR, 3.9–7.7) years, 34,872 dementia cases occurred (22,511 AD events over 6.3 years, 7075 VaD events over 6.4 years). The incidence of overall dementia was 2.96, 2.76, and 2.32 per 100 PY in the low, moderate, and high CVH status. The incidence of AD and VaD were 1.82, 1.76, and 1.55 per 100 PY, 0.61, 0.53, and 0.39 per 100 PY in those with low, moderate, and high CVH status, respectively (Tables 2 and 3). The cumulative incidence of each type of dementia was lower in those with high or moderate CVH status than in those with low CVH status (Supplementary Fig. S3).

**Table 2:** Incidence rates, absolute rate differences, and time-varying HRs for overall dementia according to CVH Status and increasing number of optimal CVH metrics and CVH scores.

Overall dementia	No. of case/total No	Incidence rate per 100 PY	ARD per 100 PY (95% CI)	Time-varying adjusted HR^a^ (95% CI)
***Before censoring stroke***				
CVH status, No. of metrics at the optimal level^b^				
Low, 0–2	15,154/89,560	2.96	0 (reference)	1 (reference)
Moderate, 3–4	18,193/116,030	2.76	− 0.19 (− 0.26 to − 0.13)	0.91 (0.89–0.92)
High, 5–6	1525/12,146	2.32	− 0.64 (− 0.76 to − 0.51)	0.78 (0.75–0.80)
Per additional CVH metric at the optimal level (range 0–6)^b^				0.94 (0.93–0.94)
Per 1-point increase in the 12-point CVH score (range 0–12)^b,c^				0.93 (0.93–0.94)
***After censoring stroke***				
CVH status, No. of metrics at the optimal level^b^				
Low, 0–2	14,000/89,560	2.79	0 (reference)	1 (reference)
Moderate, 3–4	17,055/116,030	2.63	− 0.16 (− 0.22 to − 0.10)	0.91 (0.90–0.93)
High, 5–6	1465/12,146	2.25	− 0.54 (− 0.66 to − 0.41)	0.80 (0.77–0.83)
Per additional CVH metric at the optimal level (range 0–6)^b^				0.94 (0.94–0.95)
Per 1-point increase in the 12-point CVH score (range 0–12)^b,c^				0.94 (0.93–0.94)

^a^HRs with 95% CIs were estimated by time-varying Cox proportional hazard models over a median follow-up of 6.2 years (after censoring stroke, 6.1 years) for overall dementia. HRs were adjusted for age, sex, economic status, living area, comorbidities (AF, HF, MI, coronary heart disease, PAD, anemia, CKD, hyperthyroidism, hypothyroidism, osteoporosis, sleep apnea, COPD, chronic liver disease, and cancer), medications (oral anticoagulants, antiplatelet agents), depressive mood, lower extremity function, and cognitive function at baseline.

^b^CVH status, per additional CVH metric at the optimal level and per 1-point increase in the 12-point CVH score, were used as time-varying variables.

^c^The continuous 12-point CVH score (range, higher score indicating higher CVH) was calculated by assigning 0 (poor), 1 (intermediate), and 2 (optimal) points to each of the six metrics and summing them.

*AF* atrial fibrillation, *ARD* absolute rate difference, *CI* confidence interval, *CKD* chronic kidney disease, *COPD* chronic obstructive pulmonary disease, *CVH* cardiovascular health, *HF* heart failure, *HR* hazard ratio, *MI* myocardial infarction, *PAD* peripheral artery disease, *PY* person-years.

**Table 3:** Incidence rates, absolute rate differences, and time-varying HRs for Alzheimer’s and vascular dementia according to CVH Status and increasing number of optimal CVH metrics and CVH scores.

	No. of case/total No	Incidence rate per 100 PY	ARD per 100 PY (95% CI)	Time-varying adjusted HR^a^ (95% CI)
**Alzheimer’s dementia**				
***Before censoring stroke***				
CVH status, No. of metrics at the optimal level^b^				
Low, 0–2	9598/89,560	1.82	0 (reference)	1 (reference)
Moderate, 3–4	11,875/116,030	1.76	− 0.06 (− 0.11 to − 0.01)	0.92 (0.90–0.94)
High, 5–6	1038/12,146	1.55	− 0.27 (− 0.37 to − 0.17)	0.82 (0.79–0.85)
Per additional CVH metric at the optimal level (range 0–6)^b^				0.95 (0.94–0.95)
Per 1-point increase in the 12-point CVH score (range 0–12)^b,c^				0.94 (0.93–0.94)
***After censoring stroke***				
CVH status, No. of metrics at the optimal level^b^				
Low, 0–2	9011/89,560	1.75	0 (reference)	1 (reference)
Moderate, 3–4	11,286/116,030	1.71	− 0.05 (− 0.10 to 0.00)	0.92 (0.90–0.94)
High, 5–6	1010/12,146	1.53	− 0.23 (− 0.33 to − 0.12)	0.84 (0.81–0.87)
Per additional CVH metric at the optimal level (range 0–6)^b^				0.95 (0.94–0.96)
Per 1-point increase in the 12-point CVH score (range 0–12)^b,c^				0.94 (0.94–0.95)
**Vascular dementia**				
***Before censoring stroke***				
CVH status, No. of metrics at the optimal level^b^				
Low, 0–2	3207/89,560	0.61	0 (reference)	1 (reference)
Moderate, 3–4	3609/116,030	0.53	− 0.07 (− 0.10 to − 0.05)	0.86 (0.83–0.89)
High, 5–6	259/12,146	0.39	− 0.22 (− 0.27 to − 0.17)	0.66 (0.61–0.71)
Per additional CVH metric at the optimal level (range 0–6)^b^				0.91 (0.89–0.92)
Per 1-point increase in the 12-point CVH score (range 0–12)^b,c^				0.90 (0.89–0.91)
***After censoring stroke***				
CVH status, No. of metrics at the optimal level^b^				
Low, 0–2	2747/89,560	0.53	0 (reference)	1 (reference)
Moderate, 3–4	3151/116,030	0.47	− 0.06 (− 0.08 to − 0.03)	0.87 (0.83–0.90)
High, 5–6	232/12,146	0.35	− 0.18 (− 0.23 to − 0.13)	0.68 (0.62–0.74)
Per additional CVH metric at the optimal level (range 0–6)^b^				0.91 (0.89–0.92)
Per 1-point increase in the 12-point CVH score (range 0–12)^b,c^				0.91 (0.90–0.92)

^a^HRs with 95% CIs were estimated by time-varying Cox proportional hazard models over a median follow-up of 6.3 years (after censoring stroke, 6.3 years) for Alzheimer’s dementia and 6.4 years (after censoring stroke, 6.3 years) for vascular dementia. HRs were adjusted for age, sex, economic status, living area, comorbidities (AF, HF, MI, coronary heart disease, PAD, anemia, CKD, hyperthyroidism, hypothyroidism, osteoporosis, sleep apnea, COPD, chronic liver disease, and cancer), medications (oral anticoagulants, antiplatelet agents), depressive mood, lower extremity function, and cognitive function at baseline.

^b^CVH status, per additional CVH metric at the optimal level and per 1-point increase in the 12-point CVH score, were used as time-varying variables.

^c^The continuous 12-point CVH score (range, higher score indicating higher CVH) was calculated by assigning 0 (poor), 1 (intermediate), and 2 (optimal) points to each of the six metrics and summing them.

*AF* atrial fibrillation, *ARD* absolute rate difference, *CI* confidence interval, *CKD* chronic kidney disease, *COPD* chronic obstructive pulmonary disease, *CVH* cardiovascular health, *HF* heart failure, *HR* hazard ratio, *MI* myocardial infarction, *PAD* peripheral artery disease, *PY* person-years.

In multivariable time-varying Cox regression analysis, compared with low CVH status, moderate and high CVH status were associated with lower risks of overall dementia (HR [95% confidence interval (CI)], 0.91 [0.89–0.92] for moderate; 0.78 [0.7–0.80] for high CVH status), AD (0.92 [0.90–0.94] for moderate; 0.82 [0.79–0.85] for high CVH status), and VaD (0.86 [0.83–0.89] for moderate; 0.66 [0.61–0.71] for high CVH status). The risks of overall dementia (0.94 [0.93–0.94] per additional metric; 0.93 [0.93–0.94] per 1-point increase), AD (0.95 [0.94–0.95] per additional metric; 0.94 [0.93–0.94] per 1-point increase), and VaD (0.91 [0.89–0.92] per additional metric; 0.90 [0.89–0.91] per 1-point increase) decreased with an increase in CVH metrics at the optimal level and with an increase in the 12-point CVH score. A total of 8758 participants with stroke events during the follow-up period were censored at the date of the incident stroke. After censoring for interim stroke, the associations of CVH status with the risk of dementia including AD and VaD remained significant (Tables 2 and 3).

### Risk of dementia by the number of optimal CVH metrics and the CVH score

**Figure 2:**
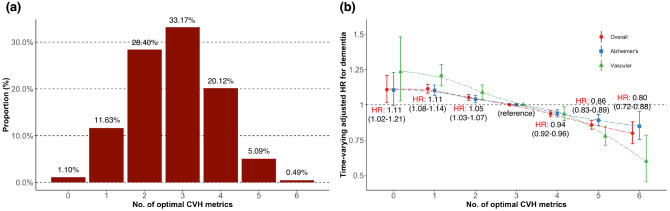
Plots of time-varying HRs for the association of the number of optimal CVH metrics with the risk of dementia. (**A**) The proportion of participants according to the number of optimal CVH metrics. (**B**) Time-varying HRs plot according to the number of optimal CVH metrics. Stroke censoring was performed in the analysis. Time-varying HRs were estimated using the group with the median optimal metrics of 3 as the reference groups. All multivariable time-varying Cox regression models were adjusted for age, sex, economic status, living area, comorbidities (AF, HF, MI, coronary heart disease, PAD, anemia, CKD, hyperthyroidism, hypothyroidism, osteoporosis, sleep apnea, COPD, chronic liver disease, and cancer), medications (oral anticoagulants, antiplatelet agents), depressive mood, lower extremity function, and cognitive function at baseline. Each type of dots with interval bars indicates time-varying multivariable-adjusted HRs and 95% CIs (exact values of HR with 95% CI on overall dementia only are displayed above or below each interval bar). Two-dashed lines indicate the trend of changes in HRs according to the number of optimal CVH metrics. Abbreviations: *AF* atrial fibrillation, *CI* confidence interval, *CKD* chronic kidney disease, *COPD* chronic obstructive pulmonary disease, *CVH* cardiovascular health, *HF* heart failure, *HR* hazard ratio, *MI* myocardial infarction, *PAD* peripheral artery disease.

In the distribution of participants according to the number of optimal CVH metrics and the CVH scores, the median and highest proportions of participants were 3 optimal CVH metrics and a CVH score of 8 (Fig. 2; Supplementary Fig. S4). When compared to 3 optimal metrics as the reference groups, time-varying adjusted HRs for each type of dementia represented an inverse proportional relationship of HRs as the number of optimal CVH metrics increases or decreases centered on the reference value (Fig. 2). A similar trend was also seen in the plot of time-varying adjusted HRs according to continuous CVH scores, except for some very low CVH scores. CVH scores of 1 and 2 (with a wide range of CI) had a lack of statistical power due to the relatively small number of participants and low event rate of dementia (Supplementary Fig. S4 in the Supplement).

### Risk of dementia by the levels of individual CVH components

**Figure 3:**
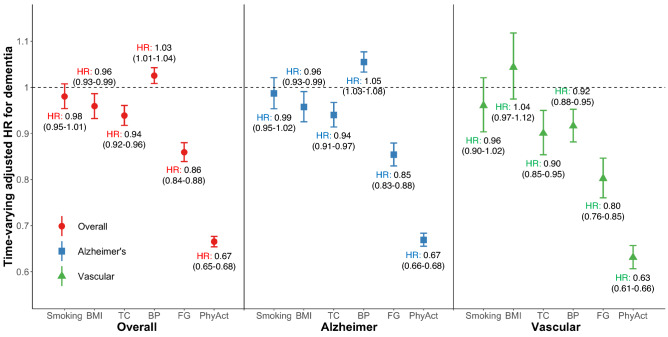
A plot of time-varying HRs for the association of individual components of CVH metrics with the risk of dementia. Stroke censoring was performed in the analysis. The associations between the risks of each type of dementia and individual components of CVH metrics were investigated separately. Each level of individual CVH metrics was included as a time-varying variable, and the poor levels of each component was used as a reference value compared to the intermediate and optimal levels combined. All multivariable time-varying Cox regression models were adjusted for age, sex, economic status, living area, comorbidities (AF, HF, MI, coronary heart disease, PAD, anemia, CKD, hyperthyroidism, hypothyroidism, osteoporosis, sleep apnea, COPD, chronic liver disease, and cancer), medications (oral anticoagulants, antiplatelet agents), depressive mood, lower extremity function, and cognitive function at baseline. Each component of CVH metrics, as time-varying variables, was mutually adjusted for each other in addition to all other covariates. Each type of dots with interval bars indicates time-varying multivariable-adjusted HRs and 95% CIs (exact values of HR with 95% CI on each type of dementia are displayed above or below each interval bar). Abbreviations: *AF* atrial fibrillation; *BMI* body mass index, *BP* blood pressure, *CI* confidence interval, *CKD* chronic kidney disease, *COPD* chronic obstructive pulmonary disease, *CVH* cardiovascular health, *FG* fasting glucose, *HF* heart failure, *HR* hazard ratio, *MI* myocardial infarction, *PhyAct* physical activity, *PAD* peripheral artery disease, *TC* total cholesterol.

Figure 3 shows time-varying adjusted HRs of the intermediate and optimal levels combined compared to the poor levels of each component of CVH for dementia risk using levels of each component as time-varying variables. Among individual CVH metrics, physical activity had the statistically strongest association with the risk of dementia, including AD and VaD (HR [95% CI], 0.67 [0.65–0.68] for overall dementia; 0.67 [0.66–0.68] for AD; 0.63 [0.61–0.66] for VaD). Interestingly, the intermediate and optimal levels of BP metric were significantly related to an increased risk of overall dementia (1.03 [1.01–1.04]) and AD (1.05 [1.03–1.07]) compared to the poor level. On the contrary, these levels were related to a decreased risk of VaD (0.92 [0.88–0.95]). The intermediate and optimal levels of total cholesterol and fasting glucose were associated with decreased risks of dementia compared to the poor level.

## Discussion

### Main findings

This study has three key findings. First, time-dependent moderate and high measures of CVH metrics were associated with lower risks of dementia including AD and VaD compared with low CVH status, even after censoring for stroke. Second, the risk of dementia tended to decrease with the increasing number of optimal CVH metrics and the increasing CVH score. Finally, among individual CVH metrics, physical activity had the strongest association with the risk of dementia.

### Association of CVH metrics as a combined risk factor for dementia risk

Given that dementia has a multifactorial etiology and CVH metrics are not mutually exclusive, dementia prevention requires multi-domain interventions targeting cardiovascular risk factors simultaneously^3,9^. To date, several studies have investigated the association of CVH metrics the risk of dementia. Some observational cohort studies, including the Framingham Heart Study Offspring cohort, the Three-City Study cohort, and the Whitehall II Study cohort reported that increased numbers of optimal CVH metrics and a higher CVH score were associated with a lower risk of dementia^9,10,17^. Although data are limited, these results were in line with findings from our study. On the other hand, a study with a population-based cohort of the INVADE trial provided inconsistent results due to the complex relationships and offset effects of BMI and BP with dementia, and the CVH summed scores were meaningless for predicting dementia^18^. Our findings support the hypothesis that maintaining optimal levels of CVH protects against all forms of dementia including AD and VaD, and complement and align with previous studies dealing with the association between better CVH and lower risk of dementia^9,10,19^. Results reported also suggest that the evaluation of CVH metrics with respect to dementia risk may be useful in older adults and the Asian population. However, this association is still controversial because of the complexity of the relationship between different impacts and mutual influences of each component of CVH on dementia^18^.

### Association of individual components of CVH for dementia risk

In our study, among individual CVH metrics, physical activity had the strongest correlation with the risk of dementia. Increasing evidence has supported the idea that physical activity is associated with a reduced risk of dementia and that exercise interventions promote cognitive function and prevent cognitive decline in dementia^20–22^. Actually, achieving > 150 min/week of moderate-intensity aerobic exercise was associated with at least a 30% lower risk of morbidity, mortality, disability, frailty, and dementia compared with being inactive^23^. Our previous study also showed that increasing levels of physical activity were associated with a gradual decrease in the risk of dementia, and even a low amount of light-intensity physical activity was associated with reduced dementia risk^24^. Our results are consistent with previous studies and highlight the importance of the role of CVH, in particular physical activity, in promoting brain health at older ages. Physical activity can be a promising intervention for the prevention and non-pharmacological treatment of dementia in that it contributes to the improvement of cognitive function.

Each behavioral and biological metric of CVH was associated with the incidence of dementia, however, the direction of their association with the risk of dementia seems to be different especially in the BP metric. Hypertension in mid-life and hypotension in late life seemed to be associated with an increased risk for subsequent dementia^25^. In another study, the risk of overall dementia and AD was significantly higher in systolic BP ≥ 160 or lower systolic BP with U-shaped associations, on the other hand, the risk of VaD increased gradually as systolic BP increased^26^. Our previous study showed among midlife AF patients, there were U-shaped relationships between systolic or diastolic BP and the risk of dementia, and according to dementia subtype, different ways of association between BP and dementia were observed: low BP correlated to a higher risk of AD, whereas high BP was associated with a higher risk of VaD^14^. Interesting findings of this study that an adequate or low level of BP was associated with an increased risk of overall dementia and AD rather than high BP are consistent with the results of the above-mentioned studies and might be due to the older study participants corresponding to late life with a mean age of 71.6 years and lower BP group which could be included in intermediate and optimal levels of BP metric.

Given the complex relationship between individual CVH metrics and dementia risk, for CVH metrics to have high predictive validity for dementia, components that show unambiguous associations with dementia should be highlighted^18^. By weighting on these CVH components, CVH metrics might offer a clear starting point for the prevention of dementia. Evaluation of differential weighting of CVH metrics and of potential interaction between CVH metrics in predicting dementia could be important in future studies^10^.

### Study limitations

The current study has limitations. First, because the CVH metrics employed in this study did not include dietary habits, this may compromise the comparability between our study and other existing studies using CVH metrics based on the 7-item tool (Life’s Simple 7) suggested by the AHA. Second, the assessment of physical activity measured using self-report techniques may be prone to induce recall bias and measurement error, although not limited to this study. Third, although large nationwide population databases are increasingly used for clinical research, such studies are potentially susceptible to errors arising from inaccuracies and the complexity of using codes. To minimize this problem, we applied the definitions that have been previously validated in our previous studies^15,16,27,28^. Despite validation studies using medical records and cognitive tests, the uncertainty of assigning a specific dementia diagnosis in clinical practice using the ICD codes still exists. Recently, the 2018 National Institute on Aging-Alzheimer's Association Research Framework suggested a framework for AD's biological definition that involves the biomarkers of amyloid and Tau^29^. In this regard, the diagnosis of dementia using the ICD-10 codes used in this study is closer to the diagnosis by clinical criteria than the biological diagnosis. Fourth, our study had a relatively short follow-up period for dementia compared to other longitudinal studies dealing with dementia risk. In general, dementia has a long preclinical period so that that the complex underlying mechanisms of dementia can take decades to lead to the development of dementia symptoms^29^. Lastly, despite adjusting for differences in baseline characteristics between the study groups, there may still be unmeasured potential confounders that may act as other significant factors for predicting dementia. Especially, educational level, genetic factors (e.g., apolipoprotein E4), occupational position or history, and marital status were used as crucial clinical variables for adjustment in other studies examining the risk of dementia by CVH^9,10,17,18^.

## Conclusions

In an older Asian population without prior dementia or cerebrovascular diseases, a consistent relationship was observed between the improvement of a composite CVH metric and a reduced risk of dementia, including both AD and VaD. Maintaining optimal levels of CVH may be helpful in lowering dementia risk and among individual components of CVH, the improvement especially in physical activity has the great potential to alleviate the public health burden of dementia.

## Supplementary Information

Supplementary Information.

## Data Availability

The data that support the findings of this study are available from the National Health Information Database, which is an online-only database for qualified analysis centers with formal payment and strict regulations on data release (https://nhiss.nhis.or.kr/). The restrictions apply to the availability of these data, which were used under license for the current study, and so are not publicly available. However, the datasets generated during and/or analyzed during the current study are available from the corresponding authors [Prof. BJ, cby6908@yuhs.ac] upon reasonable request and with permission of the NHIS.
