# Supplementary material for: Association of cardiovascular health with the risk of dementia in older adults
Source: Sci Rep. 2022 Sep 19;12:15673. doi: 10.1038/s41598-022-20072-3 (PMC9485258; doi:10.1038/s41598-022-20072-3)
Supplement: Supplementary file 1 — Supplementary Information. [file 41598_2022_20072_MOESM1_ESM.pdf]

## Supplementary information

## Association of cardiovascular health with the risk of dementia in older adults

Seunghoon Cho, MD; Pil-Sung Yang, MD; Daehoon Kim, MD; Seng Chan You, MD, MS; Jung-Hoon Sung, MD; Eunsun Jang, MS; Hee Tae Yu, MD; Tae-Hoon Kim, MD; Hui-Nam Pak, MD; Moon-Hyoung Lee, MD; Boyoung Joung, MD, PhD

**Contents**

**Supplementary Table S1.** Definitions of CVH metrics according to the American Heart Association for ascertainment of cardiovascular health status.

**Supplementary Table S2.** Definitions and ICD-10 codes used for covariates and clinical outcomes.

**Supplementary Figure S1.** Summary of the study design.

**Supplementary Figure S2.** Changes in the distribution of levels of individual CVH components according to CVH status at each health examination (1st ~ 5th check-ups).

**Supplementary Figure S3.** Cumulative incidence curves of each type of dementia according to baseline CVH status. (a) Incidence of overall dementia. (b) Incidence of Alzheimer's dementia. (c) Incidence of vascular dementia.

**Supplementary Figure S4.** Plots of time-varying HRs for the association of the continuous 12-point CVH score with the risk of dementia. (a) The proportion of participants according to the CVH scores. (b) Time-varying HRs plot according to the CVH scores.

**Supplementary Table S1. Definitions of CVH metrics according to the American Heart Association for ascertainment of cardiovascular health status.**[1]

| Metrics | Poor level (score=0) | Intermediate level (score=1) | Optimal level (score=2) |
|---|---|---|---|
| Smoking | Current smokers | Quit <12 months | Never or quit ≥12 months |
| Physical activity[a] | None | 1–74 min/week vigorous activity, 1–149 min/week moderate activity or a combination of the two | ≥75 min/week of vigorous activity, ≥150 min/week of moderate activity or a combination of the two |
| Body mass index | ≥30 kg/m$^2$ | 25-29.9 kg/m$^2$ | <25 kg/m$^2$ |
| Fasting plasma glucose[b] | ≥126 mg/dL | 100-126 mg/dL or <100 mg/dL treated | <100 mg/dL, untreated |
| Total cholesterol[b] | >240 mmol/L | 200-240 mg/dL or <200 mg/dL treated | <200 mg/dL, untreated |
| Blood pressure[c] | ≥140/90 mmHg | <120/80 mmHg on medications or 120-139/80-89 mmHg | <120/80 mmHg, untreated |

[a] Physical activity was assessed using questions on frequency and duration of participation in mildly energetic (e.g., weeding, general housework, bicycle repair), moderately energetic (e.g., dancing, cycling, leisurely swimming), and vigorous physical activity (e.g., running, hard swimming, playing squash).[2-4]

[b] SI conversions: To convert cholesterol to millimoles per liter, multiply by 0.0259; to convert glucose to millimoles per liter, multiply by 0.0555.

[c] Systolic blood pressure was measured twice with a sphygmomanometer in the sitting position after 5 min rest, and the average of the two readings was used in the present analyses.

Abbreviations: CVH, cardiovascular health.

**Supplementary Table S2. Definitions and ICD-10 codes used for covariates and clinical outcomes.**

| | Definitions | ICD-10 codes or conditions |
|---|---|---|
| **Exclusion criteria** | | |
| Ischemic stroke | Defined from diagnosis[a] | ICD-10: I63, I64 |
| Transient ischemic attack | Defined from diagnosis[a] | ICD-10: G45 |
| Hemorrhagic stroke | Defined from diagnosis[a] | ICD-10: I60, I61, I62 |
| **Comorbidities** | | |
| Atrial fibrillation | Defined from diagnosis[a] | ICD-10: I48 |
| Heart failure | Defined from diagnosis[a] | ICD-10: I11.0, I50, I97.1 |
| Myocardial infarction | Defined from diagnosis[a] | ICD-10: I21, I22, I25.2 |
| Coronary heart disease | Defined from diagnosis[a] and intervention | Acute myocardial infarction (ICD-10: I20x, I21x) and coronary angiography (HA670, HA680, HA681) |
| | Coronary revascularization | PTCA (Percutaneous Transluminal Coronary Angioplasty): M6551, M6552<br>PCI (Percutaneous Coronary Intervention; stent insertion): M6561, M6563, M6562, M6564<br>Percutaneous transluminal coronary atherectomy: M6571, M6572<br>Percutaneous thrombus removal, Thrombolytic treatment: M6634<br>Coronary artery bypass graft: O1641, OA641, O1642, OA642, O1647, OA647 |
| | Defined from diagnosis[a] | I25.2 (Old myocardial infarction), I25.5 (Ischemic cardiomyopathy), I25.6 (Silent myocardial ischemia), I25.8 (Myocardial infarction as chronic), I25.9 (Chronic ischemic heart disease) |
| Peripheral artery disease | Defined from diagnosis[a] | ICD-10: I70.0, I70.1, I70.2, I70.8, I70.9 |
| Anemia | Defined from laboratory data<br>(if laboratory data were available, it was defined) | Hemoglobin concentration <13g/dL in men and <12g/dL in women |
| Chronic kidney disease | Defined from eGFR or diagnosis[a]<br>(if laboratory value was not available, diagnosis code was used) | eGFR <60mL/min per $1.73m^2$<br>ICD-10: N18, N19 |
| Hyperthyroidism | Defined from diagnosis[a] | ICD-10: E05 |
| Hypothyroidism | Defined from diagnosis[a] | ICD-10: E03 |
| Osteoporosis | Defined from diagnosis[a] | ICD-10: M80, M81, M82 (except |

| | | |
|---|---|---|
| | | M82.0) |
| Sleep apnea | Defined from diagnosis[a] | ICD-10: G47.3 |
| Chronic obstructive pulmonary disease | Defined from diagnosis[a] plus treatment | ICD-10: J42, J43 (except J43.0), J44<br>Treatment: SABA, SAMA, LABA, LAMA, ICS, ICS+LABA, or methylxanthine (>1 months) |
| Chronic liver disease | Defined from diagnosis[a] of chronic liver disease, cirrhosis, and hepatitis | ICD-10: B18, K70, K71, K72, K73, K74, K76.1 |
| Cancer | Defined from diagnoses[a] of cancer (non-benign) | ICD-10: C00-C97 |
| **Clinical outcomes** | | |
| (Overall) Dementia | Defined from diagnosis with prescription of dementia drugs | ICD-10: F00, G30 (Alzheimer's dementia), F01 (Vascular dementia), F02 (Dementia with other diseases classified elsewhere), F03 or G31 (Unspecified dementia)<br>Dementia drugs: rivastigmine, galantamine, memantine, or donepezil |
| Alzheimer's disease | Defined from diagnosis with prescription of dementia drugs | ICD-10: G30<br>Dementia drugs: rivastigmine, galantamine, memantine, or donepezil |
| Vascular dementia | Defined from diagnosis with prescription of dementia drugs | ICD-10: F01<br>Dementia drugs: rivastigmine, galantamine, memantine, or donepezil |
| **Other covariates** | | |
| Economic status | Determined on the basis of the relative economic levels categorized into 10 levels according to the total amount of national health insurance premiums paid by the insured individual in the index year, proportional to the individual's income. Economic status "Middle" denotes economic level 5~7, or the middle third of income, and "High" denotes economic level 8 or higher, or the top third of income among the entire Korean population. | |
| Living area | Determined on the basis of the address code currently living in (Small city or rural area/Metropolitan city) | |

[a] To ensure accuracy, comorbidities were established based on one inpatient or two outpatient records of ICD-10 codes in the database.

Abbreviations: eGFR, estimated glomerular filtration rate; ICD-10, international classification of diseases-10th revision.

**Supplementary Figure S1. Summary of the study design.**

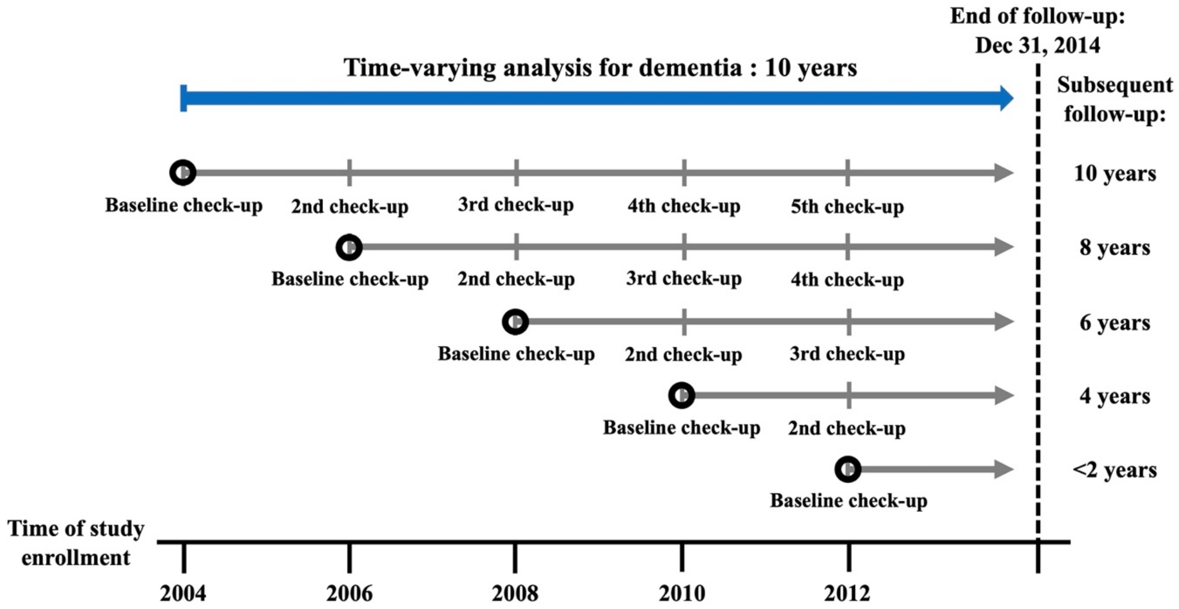


The first vertical lines (marked as "Baseline check-up") of each gray line present the time of study enrollment of participants with a baseline health examination. The rest of the vertical gray lines after baseline check-up present the time of additional health examinations during the follow-up period. The timing of study registration was different for each participant, so the subsequent follow-up period and the number of additional health examinations received during this period were different (participants first enrolled in 2004 received baseline examination plus 4 additional health examinations, while participants last enrolled in 2012 did not receive additional examinations until the follow-up ended, December 31, 2014).

**Supplementary Figure S2. Changes in the distribution of levels of individual CVH components according to CVH status at each health examination (1st ~ 5th check-ups).**

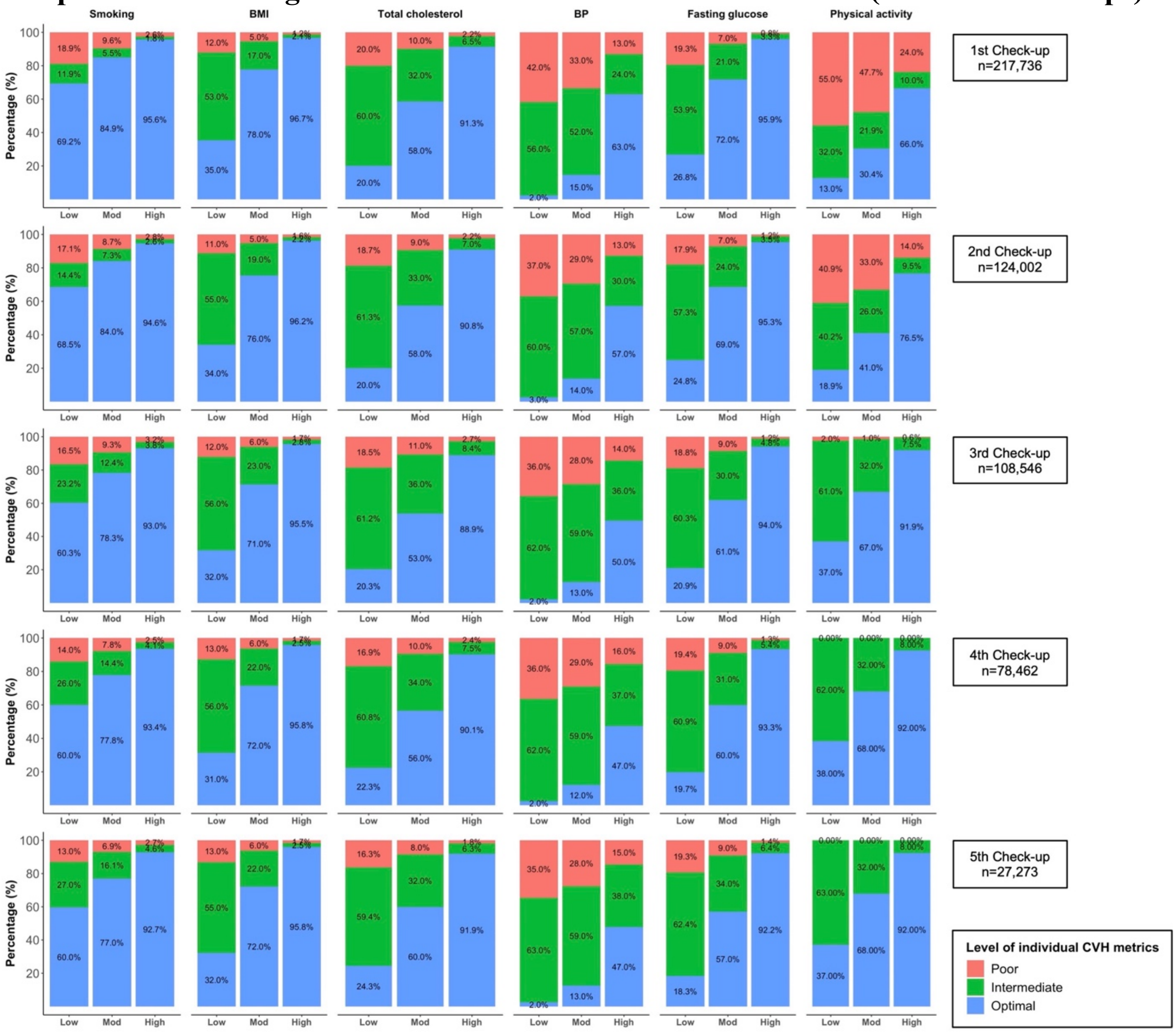


Percentage (%) denotes the proportion of levels of each component of CVH metrics according to CVH status.

Abbreviations: BMI, body mass index; BP, blood pressure; CVH, cardiovascular health; Mod, moderate.

**Supplementary Figure S3. Cumulative incidence curves of each type of dementia according to baseline CVH status. (a) Incidence of overall dementia. (b) Incidence of Alzheimer's dementia. (c) Incidence of vascular dementia.**

**(a)**

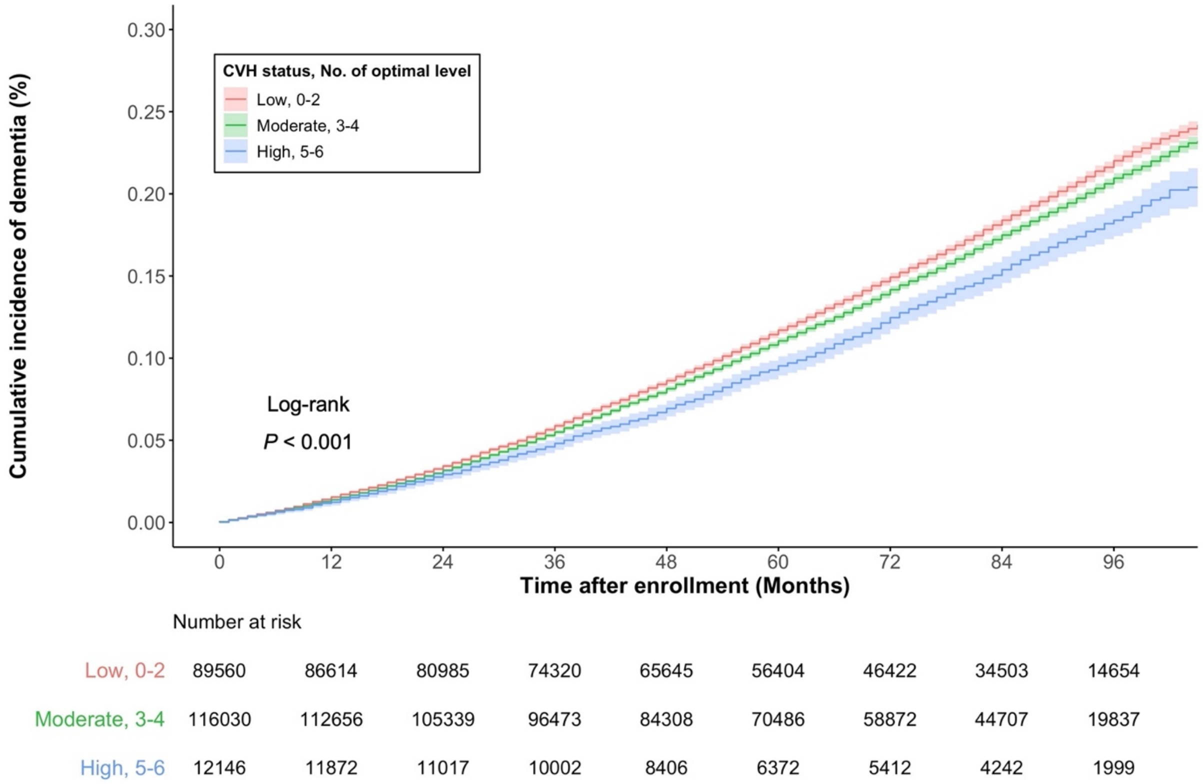


**(b)**

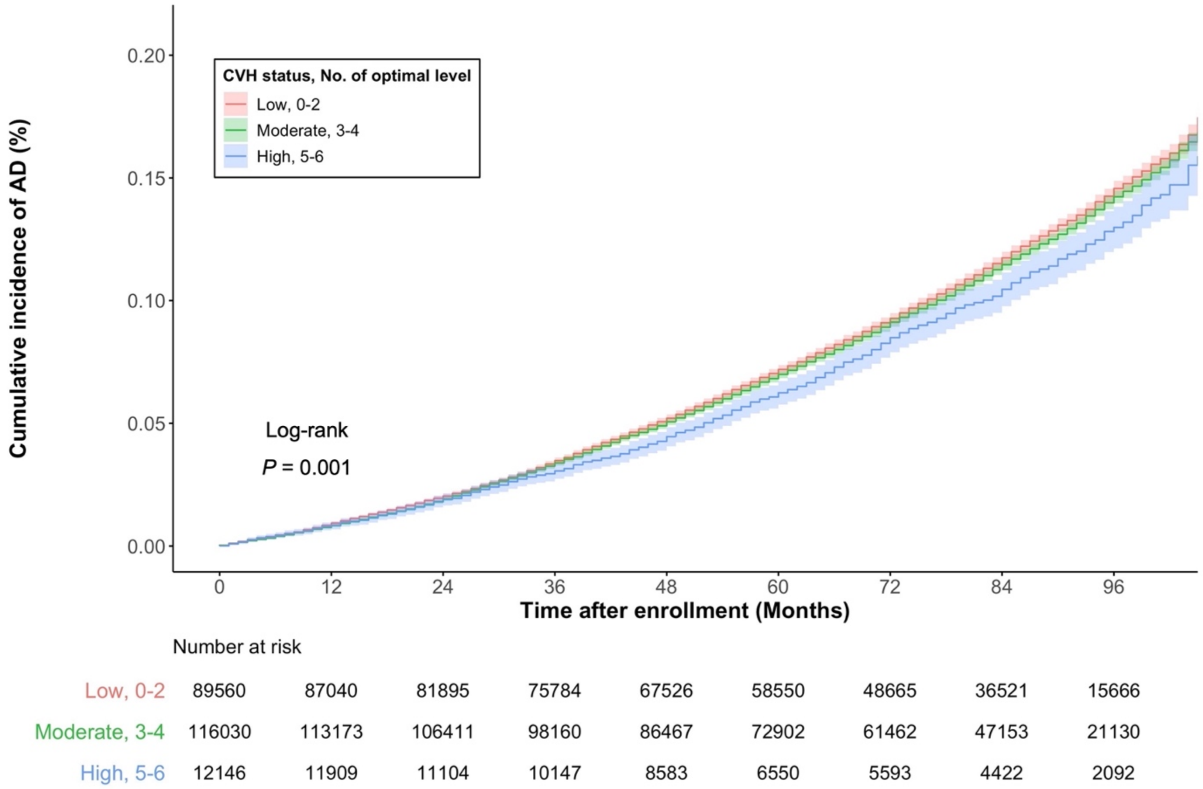


**(c)**

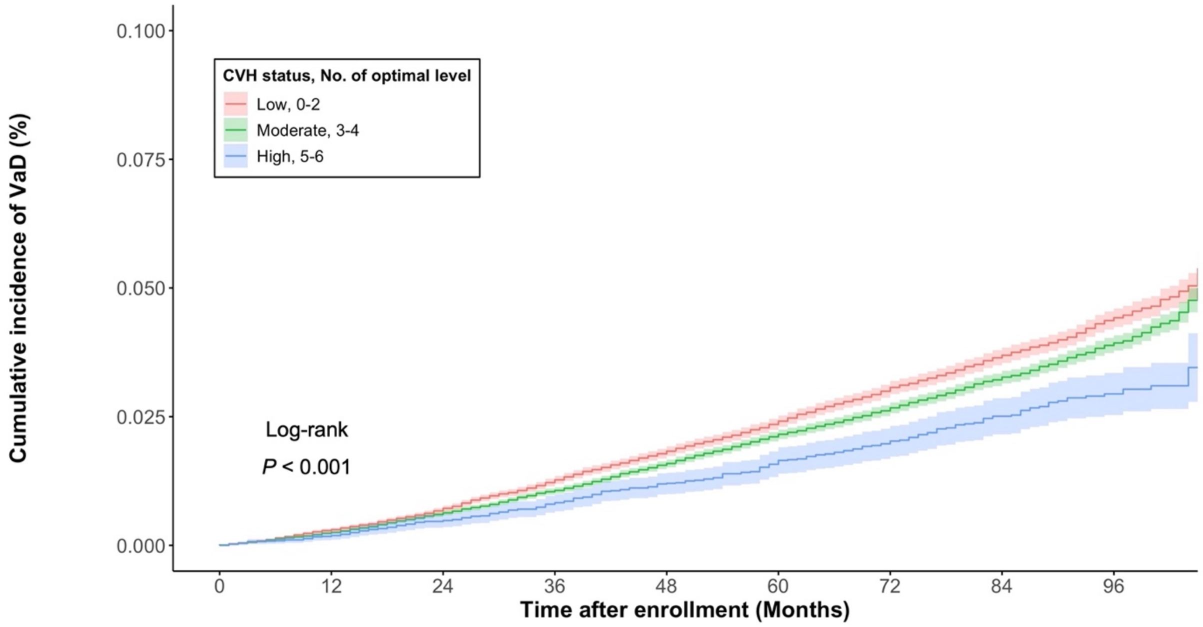


Number at risk

| | | | | | | | | | |
|---|---|---|---|---|---|---|---|---|---|
| Low, 0-2 | 89560 | 87213 | 82158 | 76167 | 67999 | 59086 | 49169 | 36965 | 15838 |
| Moderate, 3-4 | 116030 | 113405 | 106760 | 98710 | 87151 | 73605 | 62182 | 47825 | 21488 |
| High, 5-6 | 12146 | 11939 | 11157 | 10210 | 8629 | 6595 | 5653 | 4474 | 2129 |

Stroke censoring was performed in the analysis. The shaded regions of each curve indicate 95% CIs.

Abbreviations: AD, Alzheimer's dementia; CI, confidence interval; CVH, cardiovascular health; VaD, vascular dementia.

**Supplementary Figure S4. Plots of time-varying HRs for the association of the continuous 12-point CVH score with the risk of dementia. (a) The proportion of participants according to the CVH scores. (b) Time-varying HRs plot according to the CVH scores.**

**(a)**

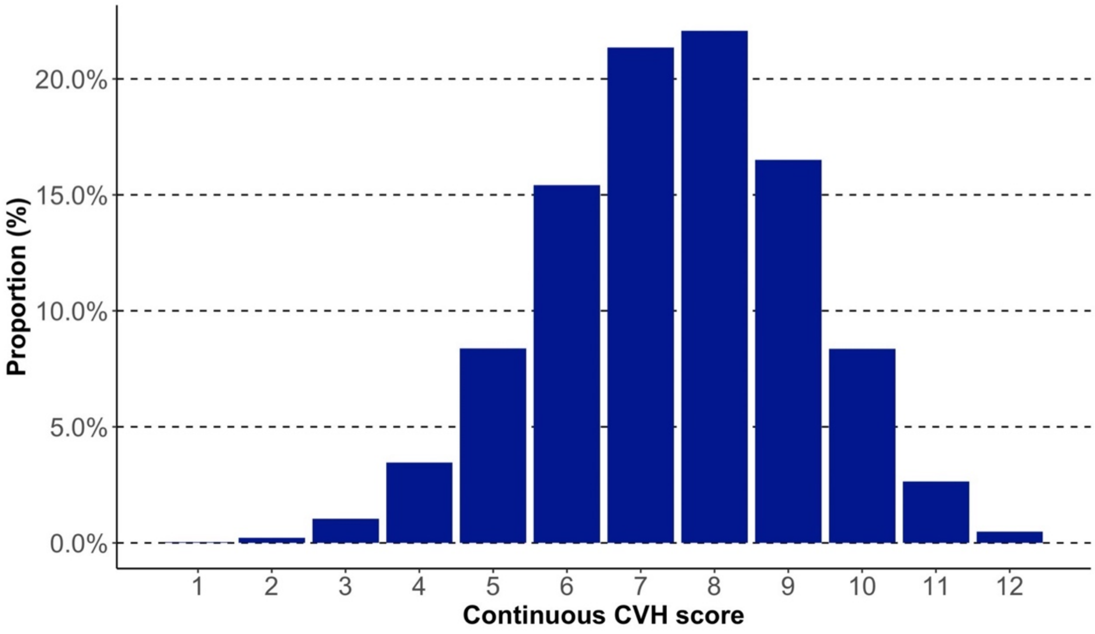


**(b)**

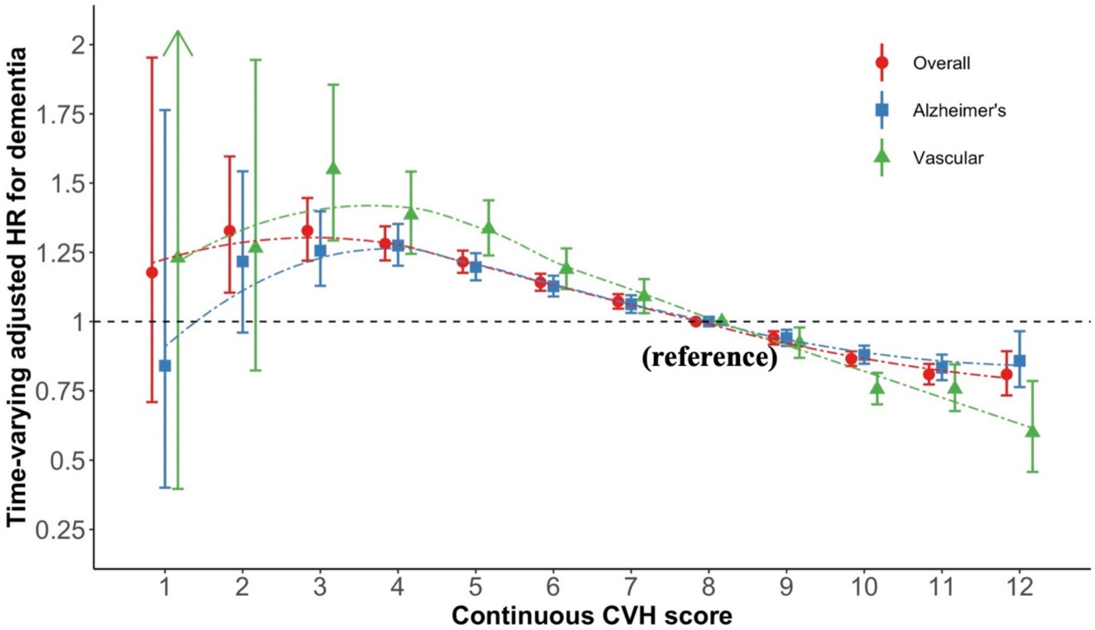


Stroke censoring was performed in the analysis. Time-varying HRs were estimated using the group with the median CVH score of 8 as the reference groups. The group with a CVH score of 0 was excluded from the analysis because of relatively very small group size (n=4) and no event of dementia. All multivariable time-varying Cox regression models were adjusted for age, sex, economic status, living area, comorbidities (AF, HF, MI, coronary heart disease, PAD, anemia, CKD, hyperthyroidism, hypothyroidism, osteoporosis, sleep apnea, COPD, chronic liver disease, and cancer), medications (oral anticoagulants, antiplatelet agents), depressive mood, lower extremity function, and cognitive function at baseline. Each type of dots with interval bars indicates time-varying multivariable-adjusted HRs and 95% CIs. Two-dashed lines indicate the trend of changes in HRs according to the CVH scores. The arrowhead

indicates the upper limit of CI extends outside the upper bound of the y-axis of the graph.
Abbreviations: AF, atrial fibrillation; CI, confidence interval; CKD, chronic kidney disease; COPD, chronic obstructive pulmonary disease; CVH, cardiovascular health; HF, heart failure; HR, hazard ratio; MI, myocardial infarction; PAD, peripheral artery disease.

**References**

1. Lloyd-Jones, D. M. *et al.* Defining and setting national goals for cardiovascular health promotion and disease reduction: the American Heart Association's strategic Impact Goal through 2020 and beyond. *Circulation.* **121**, 586-613 (2010).

2. Richardson, M. T., Leon, A. S., Jacobs, D. R., Jr., Ainsworth, B. E. & Serfass, R. Comprehensive evaluation of the Minnesota Leisure Time Physical Activity Questionnaire. *J Clin Epidemiol.* **47**, 271-281 (1994).

3. Sabia, S. *et al.* Physical activity, cognitive decline, and risk of dementia: 28 year follow-up of Whitehall II cohort study. *BMJ.* **357**, j2709 (2017).

4. van Sloten, T. T. *et al.* Association of Change in Cardiovascular Risk Factors With Incident Cardiovascular Events. *JAMA.* **320**, 1793-1804 (2018).
